# Predictors of early treatment discontinuation in patients enrolled on Phase I oncology trials

**DOI:** 10.18632/oncotarget.2909

**Published:** 2015-02-17

**Authors:** David M. Hyman, Anne A. Eaton, Mrinal M. Gounder, Erika G. Pamer, Jasmine Pettiford, Richard D. Carvajal, S. Percy Ivy, Alexia Iasonos, David R. Spriggs

**Affiliations:** ^1^ Developmental Therapeutics, Department of Medicine, Memorial Sloan Kettering Cancer Center, New York, NY 10065, USA; ^2^ Department of Epidemiology and Biostatistics, Memorial Sloan Kettering Cancer Center, New York, NY 10065, USA; ^3^ The National Cancer Institute, Bethesda, MD 20892, USA; ^4^ Weill Cornell Medical College, New York, NY 10065, USA

**Keywords:** Phase I trials, Early Discontinuation, Drug Development

## Abstract

**Purpose:**

Patients who do not complete one cycle of therapy on Phase I trials for reasons other than dose limiting toxicity (DLT) are considered inevaluable for toxicity and must be replaced.

**Methods:**

Individual records from patients enrolled to NCI-sponsored Phase I trials activated between 2000 and 2010 were used. Early discontinuation was defined as the failure to begin cycle 2 for reasons other than a DLT during cycle 1. A multinomial logistic regression with a 3-level nominal outcome (early discontinuation, DLT during cycle 1, and continuation to cycle 2) was used with continuation to cycle 2 serving as the reference category. The final model was used to create two risk scores. An independent external cohort was used to validate these models.

**Results:**

Data from 3079 patients on 127 Phase I trials were analyzed. ECOG performance status (1, ≥ 2, two-sided *P* = .0315 and *P* = .0007), creatinine clearance (<60 ml/min, *P* = .0455), alkaline phosphatase (>2.5xULN, *P* = .0026), AST (>ULN, *P* = .0076), hemoglobin (<10 g/dL, *P* < .0001), albumin (< 3.5 g/dL, *P* < .0001), and platelets (<400x109/L, *P* = .0732) were predictors of early discontinuation. The c-index of the final model was 0.63.

**Conclusion:**

Knowledge of risk factors for early treatment discontinuation in conjunction with clinical judgment can help guide Phase I patient selection.

## INTRODUCTION

Phase I eligibility criteria are intended to select fit patients with good performance status, near normal organ function, and minimal co-morbidities in order to accurately characterize the toxicity of the investigational drug [1–5]. In addition, patients with poor performance status or compromised organ function may be at increased risk for exacerbation of cancer symptoms, rapid disease progression, and death, thus necessitating discontinuation of protocol treatment during the first cycle [6–11]. Patient satisfaction with the Phase I experience may be adversely impacted by early discontinuation and their participation may delay implementation of more appropriate palliative care services [12–14]. Morever, patients who do not complete at least one cycle of therapy on Phase I trials for reasons other than dose limiting toxicity (DLT) are typically considered inevaluable for toxicity and must therefore be replaced [15]. Early discontinuation leads to delays in dose escalation as cohorts are backfilled and adds additional costs to the conduct of these studies.

Unfortunately, early trial discontinuation remains a fairly common event in Phase I studies despite strict entry criteria. In one large series, 16% of patients enrolled to Phase I trials discontinued protocol therapy within the first 21 days of beginning treatment [7]. Although prognostic risk scores are available to estimate the 90-day overall survival of Phase I trial participants, little is known about what factors put patients at risk for early trial discontinuation. No risk score or predictive model is available to estimate the risk of early discontinuation among Phase I eligible patients. In order to address this unmet need, we analyzed the individual patient records from a large cohort of 3079 patients enrolled to 127 National Cancer Institute (NCI)-sponsored Phase I clinical trials from 67 institutions throughout North America with the aim of identifying risk factors and developing a risk score for early discontinuation.

## METHODS

### Study design and patient eligibility

A multicenter cohort of all patients treated on NCI-sponsored [16] Phase I trials activated between 2000 and 2010 who met the following inclusion and exclusion criteria was used for model and risk score derivation. Data were provided from the Clinical Trials Monitoring System (CTMS) database, which is managed by Theradex Systems, Inc. The CTMS database is prospectively maintained with robust data management and auditing practices [17]. Trials of vaccines, immunotherapy, radiation therapy, loco-regional therapies, and autologous or allogeneic stem cell transplant were excluded. Organ dysfunction studies were also excluded. Eligible patients were adults (≥ 18 years) with solid tumors excluding lymphoma. Patients were required to meet a common set of Phase I laboratory criteria as follows: absolute neutrophil count (ANC) ≥ 1 × 10^9^/L, hemoglobin ≥ 8 g/dL, platelet count ≥ 75 × 10^9^/L, aspartate aminotransferase (AST) or alanine aminotransferase (ALT) ≤ 5x upper limit of normal (ULN), and total bilirubin ≤ 2x ULN. This led to the exclusion of 5% of patients in the derivation dataset, reducing the number of patients from 3910 to 3717. Patients with incomplete data for one or more of the covariates included in the final multivariate model were also excluded. On this basis, 17% (*N* = 638) of the 3717 patients in the derivation set were excluded. Baseline characteristics of patients with complete and missing data were largely similar (data not shown). All patients had regular follow-up visits as specified by the protocol to which they were enrolled. Patients must have received at least one dose of study drug(s) to be included in the analysis.

An independent cohort of 232 patients consecutively enrolled to 20 Phase I trials between 2009 and 2012 in the Developmental Therapeutics Clinic at Memorial Sloan Kettering Cancer Center was used as a validation set. CTEP-sponsored studies included in the derivation set were excluded from this cohort. Patients and trials in the validation set were required to meet the same eligibility criteria as the derivation set.

### Outcome

The primary outcome for each patient was early discontinuation (yes/no), which was defined as: 1) the inability to begin cycle 2 of therapy, and 2) the absence of any dose limiting toxicity (DLT) during cycle 1. The DLT criteria were standardized to allow for a uniform outcome definition across all trials and were defined as a grade ≥ 3 non-hematologic or grade ≥ 4 hematologic toxicity attributed as at least possibly related to study treatment, excluding asymptomatic electrolyte abnormalities. Toxicity level data were used to determine whether each patient had an adverse event that qualified under this definition of DLT. These DLT criteria are commonly used in contemporary Phase I studies [15, 18].

### Candidate factors

The following categories of baseline patient characteristics were investigated for their association with early trial discontinuation: 1) commonly utilized Phase I eligibility criteria, 2) established prognostic factors, 3) prior treatment exposure, and 4) disease burden.

### Model and risk score building and validation

All variables except ECOG performance status (PS) were dichotomized to facilitate the final goal of creating a simple risk score. Cutoffs were chosen based on upper limit of normal and/or cutoffs defined by the NCI Common Terminology Criteria of Adverse Events (CTCAE), most commonly using the criteria for grade 1 abnormalities. For certain covariates, grade 1 abnormalities were very common and would therefore have been of limited discriminatory utility and in those cases criteria for grade 2 abnormalities were utilized. Similarly, cutoffs that would identify a small minority of patients and thus have limited clinical utility and statistical properties were avoided. ECOG PS was treated as a categorical variable with three levels: 0, 1, and ≥ 2. Due to concern about the heterogeneity of the patients who did not discontinue early (this group contains both patients who experienced a DLT during cycle 1 and patients who began cycle 2 without a DLT), multinomial logistic regression with a 3-level nominal outcome (early discontinuation as defined above, DLT during cycle 1, and continuation to cycle 2) was used to assess relationships between factors and outcome. Continuation to cycle 2 was the reference category.

Candidate factors for the multivariate model were selected based on clinical reasoning and univariate results, and the final model was selected using a backward selection procedure where variables with the largest discontinuation-specific *P*-value (ie, the *P*-value associated with the odds ratio for early discontinuation versus continuation to cycle 2) were sequentially removed until all discontinuation-specific *P*-values were < 0.10. The final logistic model assigns each patient a predicted risk of cycle 1 DLT, early discontinuation, and continuation to cycle 2. The binary outcome for model validation was early discontinuation (yes/no), with cycle 1 DLT and continuation to cycle 2 combined. Predictive accuracy of the multinomial logistic model was evaluated with the concordance-index (c-index), which estimates the proportion of pairs where the patient who discontinued early has a higher model-predicted risk of early discontinuation than the patient who did not discontinue early.

The final multinomial model (based on Hosmer equations 8.1 to 8.5 [19]) was used to create two risk scores. In the expanded risk score, points were assigned to each risk factor in order to best preserve the ranking of the patients’ risk of early discontinuation assigned by the full model, while ensuring that the model was not overly complex [20]. A simplified risk score was subsequently developed in which only the strongest factors (those assigned 2 points in the expanded risk score) contribute one point each. Ninety-five percent confidence intervals for sensitivity, specificity, and overall correct classification rate were calculated using exact confidence intervals for binomial proportions.

The risk scores were dichotomized at various cutoff points to define patients at low and high risk of early discontinuation. Overall correct classification rate was defined as the percent of patients who were classified correctly: either as low risk and did not discontinue early, or as high risk and did discontinue early. Two cutoffs (5 for the expanded risk score, and 2 for the simplified risk score) were selected based on their performance in the CTEP data, with an emphasis on high specificity, which translates to a low probability of classifying patients who did not discontinue early as high risk. These were then validated on an external dataset. C-index, sensitivity, specificity, and overall correct classification rate were used to assess full model performance and risk score performance in the external data.

All statistical analysis was performed in SAS 9.2 (SAS Institute, Cary, NC) and R 3.0.1 (R Foundation, Vienna, Austria) using the pROC and verification packages. All *P*-values were two-sided, and *P*-values less than 0.05 were considered significant.

## RESULTS

### Patient characteristics, derivation set

Data on 3079 patients treated on 127 Phase I trials were analyzed. Baseline patient characteristics are presented in Table 1. A broad range of tumor types was represented. The median number of prior systemic therapies was 3 (range, 0–19). Median values of pretreatment hemoglobin, platelets, calculated creatinine clearance, AST, and ALT were all within the range of normal (data not shown). In total, 508 (16.5%) patients met criteria for early discontinuation. Sixty-one percent (*N* = 312) discontinued early for clinical progression or death, 23% (*N* = 115) for an adverse event that was not a DLT, and 2% (*N* = 10) for other reasons. No off study reason was provided for 14% (*N* = 71) of the patients who discontinued early.

**Table 1:** Patient characteristics, derivation set (*N* = 3079)

Characteristic	No.	%
**Age**	58 (median)	18–87 (range)
**Sex**		
Male	1528	50%
Female	1551	50%
**Primary Tumor Site**		
Gastrointestinal	1060	34%
Genitourinary	371	12%
Thoracic	366	12%
Breast	349	11%
Gynecologic	291	9%
Sarcoma	242	8%
Head and Neck	197	6%
Melanoma and Skin	162	5%
Brain and Unknown	41	1%
**ECOG performance status**		
0	885	29%
1	2040	66%
≥ 2	154	5%
Number of Prior Systemic Therapies*		
0–2	1374	45%
3	555	18%
≥ 4	1150	37%
Prior Radiation*		
Yes	1433	47%
No	1641	53%
Number of Metastatic Sites*		
0	145	5%
1	525	18%
2	590	21%
> 2	1588	56%
Metastatic Sites (231 missing)*^1^		
Lung	1101	39%
Liver	1081	38%
Lymph Node	609	21%
Bone	216	8%
Brain	8	0%
Sum, Longest Tumor Dimensions (cm) (271 missing)*	8.1 (median)	0 – 49.5 (range)
BMI (kg/m^2^)*		
< 18.5	101	3%
≥ 18.5	2961	97%
**Laboratories**		
WBC (10^9^/L) ≥ 10.5 (2 missing)*	329	11%
ALC (10^9^/L) < 0.5 (77 missing)*	187	6%
Hemoglobin (g/dL) < 10	273	9%
Platelets (10^9^/L) ≥ 400	405	13%
Platelets (10^9^/L) < 150	230	7%
Albumin (g/dL) < 3.5	802	26%
AST (units/L) > ULN	788	26%
ALT (units/L) > ULN (92 missing)*	588	17%
Total bilirubin (mg/dL) > 1 (8 missing)*	151	5%
Alkaline Phosphatase (units/L) > 2.5xULN	355	12%
Creatinine clearance(mL/min)^2^ < 60	380	12%
**Pain at Baseline**		
Yes	208	7%
No	2871	93%

1 Patients may fall into more than one category for these covariates.

2 Estimated by Cockcroft-Gault equation, capped at 125 mL/min.

* Some patients were missing this covariate and were excluded.

### Model building and validation

**Figure 1:**
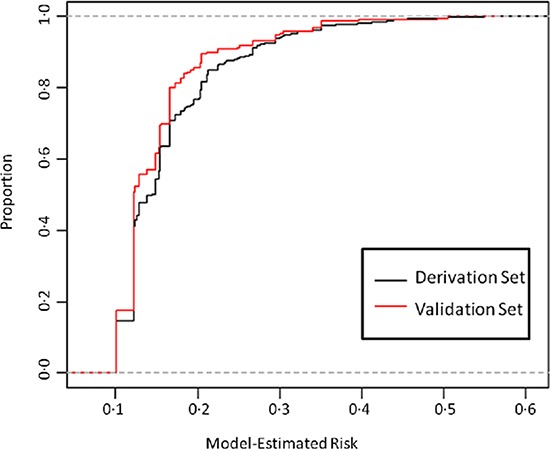
Cumulative distribution of multivariate model-estimated risk, derivation and validation set The black line represents the proportion of patients in the derivation set with an estimated risk at or below a given risk (x-axis). The red line represents the proportion of patients in the validation set with an estimated risk at or below a given risk (x-axis).

The association of baseline patient characteristics to the likelihood of early trial discontinuation was examined in the derivation set by both univariate and multivariate analysis. The results of these analyses are presented in Table 2. On univariate analysis, higher ECOG PS (*P* = 0.0008 and *P* < .0001 for PS 1, ≥ 2 respectively), WBC (*P* = 0.0005), platelet count (*P* < .0001), AST (*P* < .0001), alkaline phosphatase (*P* < .0001), ALT (*P* = 0.0305), number of prior lines of therapy (*P* = 0.0554, *P* = 0.0063 for 3, ≥ 4 respectively) and lower hemoglobin (*P* < .0001), creatinine clearance (*P* = 0.0289), number of metastatic sites (*P* = 0.0753, *P* = 0.0421 and *P* = 0.0076 for 1, 2 and ≥ 3 respectively), and albumin (*P* < .0001) were significantly associated with the risk of early discontinuation. Primary tumor site was not considered a candidate for the multivariate model because we did not wish to discriminate against specific tumor types. Similarly, prior lines of therapy and number of metastatic sites were not candidates for the final model because we did not wish to exclude broad groups of patients who constitute a significant proportion of the Phase I eligible population from participation in these studies. Other significant variables were carried forward to a multivariate model, where WBC, ALT, and absolute lymphocyte count (ALC) were not independent predictors of early discontinuation (*P* > 0.10) and were subsequently removed from the model, yielding the final model. The final multivariate model, accounting for ECOG PS (1, ≥ 2, *P* = 0.0315 and *P* = 0.0007 respectively), creatinine clearance (< 60 ml/min, *P* = 0.0455), alkaline phosphatase (> 2.5xULN, *P* = 0.0026), AST (> ULN, *P* = 0.0076), hemoglobin (< 10 g/dL, *P* < .0001), albumin (< 3.5 g/dL, *P* < .0001), and platelet count (< 400 × 10^9^/L, *P* = 0.0732) had a c-index of 0.63 in the derivation set. The c-index of this model with all the covariates included as continuous except ECOG PS was 0.64, and the c-index of our final model with the additional covariates number of prior lines of treatment and number of metastatic sites is 0.64; thus we feel that our modeling choices did not lead to decreased model performance. The odds ratios for both outcomes of the final multinomial model are shown in Supplemental Table 1A. The performance of the final multivariate model was also assessed in an independent external validation set (*N* = 232). The rate of early discontinuation in the validation set was 15%. The derivation and validation sets were well balanced with regards to the presence of factors independently associated with the risk of early discontinuation (see Supplemental Table 2A). The cumulative distribution of predicted risk of early discontinuation in the derivation and external validation sets was also similar, as demonstrated in Figure 1. The c-index of the final model in the external validation set was 0.61.

**Table 2:** Univariate and multivariate analysis, derivation set

Factor	Univariate OR^1^ (95% CI)	*P* value	Multivariate OR (95% CI)	*P* value
**ECOG**				
0	Ref	-	Ref	-
1	1.49 (1.18–1.88)	0.0008	1.30 (1.02–1.65)	0.0315
≥ 2	3.23 (2.07–5.03)	< .0001	2.22 (1.40–3.53)	0.0007
**Albumin (g/dL)**				
< 3.5	2.22 (1.79–2.74)	< .0001	1.59 (1.26–2.00)	< .0001
≥ 3.5	Ref	-	Ref	-
**Alkaline Phosphatase (units/L)**				
≤ 2.5xULN	Ref	-	Ref	-
> 2.5xULN	2.43 (1.84–3.20)	< .0001	1.62 (1.18–2.22)	0.0026
**AST (units/L)**				
≤ ULN	Ref	-	Ref	-
> ULN	1.69 (1.36–2.10)	< .0001	1.39 (1.09–1.77)	0.0076
**Creatinine clearance (mL/min)**				
< 60	1.38 (1.03–1.83)	0.0289	1.35 (1.01–1.81)	0.0455
≥ 60	Ref	-	Ref	-
**Hemoglobin (g/dL)**				
< 10	2.79 (2.09–3.72)	< .0001	1.99 (1.46–2.71)	< .0001
≥ 10	Ref	-	Ref	-
**Platelets (10^9^/L)**				
< 400	Ref	-	Ref	-
≥ 400	1.80 (1.39–2.35)	< .0001	1.30 (0.98–1.72)	0.0732
**Platelets (10^9^/L)**				
< 150	1.26 (0.87–1.81)	0.2790	NA	NA
≥ 150	Ref	-		
**ALT (units/L)**				
≤ ULN	Ref	-	NA	NA
> ULN	1.31 (1.03–1.67)	0.0305		
**WBC (10^9^/L)**				
< 4	1.27 (0.82–1.97)	0.2790	NA	NA
≥ 4	Ref			
**WBC (10^9^/L)**				
< 10.5	Ref	-	NA	NA
≥ 10.5	1.68 (1.25–2.25)	0.0005		
**ALC (10^9^/L)**			
< 0.5	1.47 (0.99–2.16)	0.0538	NA	NA
≥ 0.5	Ref	-		
**Number of Metastatic Sites**				
0	Ref	-	NA	NA
1	0.66 (0.41–1.04)	0.0753		
2	0.62 (0.40–0.98)	0.0421		
≥ 3	0.56 (0.37–0.86)	0.0076		
**Sum. Longest Tumor Dimensions (cm)**				
≤ 8	Ref	-	NA	NA
> 8	1.06 (0.86–1.30)	0.6121		
**BMI (kg/m^2^)**				
< 18.5	1.59 (0.98–2.60)	-	NA	NA
≥ 18.5	Ref	0.0616		
**Primary Site**				
Brain	1.04 (0.21–5.17)	0.9669	NA	NA
Breast	1.07 (0.78–1.49)	0.7541		
Gastrointestinal	Ref	-		
Genitourinary	0.58 (0.40–0.83)	0.0028		
Gynecologic	0.65 (0.45–0.95)	0.0266		
Head and neck	0.68 (0.43– 1.06)	0.0847		
Melanoma and skin	0.74 (0.46–1.19)	0.2120		
Sarcoma	0.68 (0.46–1.01)	0.0563		
Thoracic	0.99 (0.72–1.37)	0.9729		
Unknown	0.83 (0.27–2.52)	0.7395		
**Pain at Baseline**				
No	Ref	-	NA	NA
Yes	0.72 (0.47–1.10)	0.1287		
**Prior Lines of Systemic Therapy**				
0–2	Ref	-	NA	NA
3	1.31 (0.99–1.42)	0.0554		
≥ 4	1.36 (1.09–1.69)	0.0063		

1 Odd-ratios are for early discontinuation with continuation to cycle 2 as the reference category.

### Risk score performance

In order to create a user-friendly clinical decision aid, two risk scores were developed utilizing the final multivariate model and assessed for performance. The two proposed risks scores and their associated sensitivity, specificity, and overall correct classification rate (OCCR) in both the derivation and validation sets are shown in Table 3. An expanded risk score assigns 2 points each for: ECOG PS (≥ 2), alkaline phosphatase (> 2.5xULN), hemoglobin (< 10 g/dL), and albumin (< 3.5 g/dL), and 1 point each for: ECOG PS (= 1), creatinine clearance (< 60 ml/min), AST (> ULN), and platelets (> 400 × 10^9^/L). A simplified risk score further condenses the multivariate model by assigning 1 point each for the four characteristics with the largest impact on the risk of early discontinuation: ECOG PS (≥ 2), albumin (< 3.5 g/dL), alkaline phosphatase (> 2.5xULN), and hemoglobin (< 10 g/dL).

**Table 3:** Diagnostic accuracy of risk scores, derivation and validation sets

Expanded Risk Score^1^ 2 Points Each - ECOG 2, Alkaline Phosphatase ≥ 2.5xULN, Hemoglobin ≤ 10, and Albumin ≤ 3.5 1 Point Each - ECOG 1, Creatinine Clearance ≤ 60, AST ≥ ULN, Platelets ≥ 400			
Simplified Risk Score^2^ 1 Point Each: ECOG 2, Alkaline Phosphatase ≥ 2.5xULN, Hemoglobin ≤ 10, and Albumin ≤ 3.5			
		Derivation Set	Validation Set
**Expanded Risk Score:** < 5 points vs ≥ 5 points	Sensitivity (95% CI)	119/508 = 23.4% (19.8 – 27.4)	3/34 = 8.8% (1.9 – 23.7)
	Specificity (95% CI)	2314/2571 = 90% (88.8–91.1)	182/198 = 92% (87.2 – 95.3)
	OCCR (95% CI)	79% (77.5 – 80.5)	79.7% (74.0 – 84.7)
**Simplified Risk Score**: < 2 points vs ≥ 2 points	Sensitivity (95% CI)	113/508 = 22.2% (18.7 – 26.1)	4/34 = 11.8% (3.3 – 27.5)
	Specificity (95% CI)	2317/2571 = 90.1% (88.9–91.3)	182/198 = 91.9% (87.2 – 95.3)
	OCCR (95% CI)	78.9% (77.4 – 80.4)	80.2% (74.5 – 85.1)

1 Maximum possible score: 11

2 Maximum possible score: 4

OCCR: Overall Correct Classification Rate.

**Figure 2:**
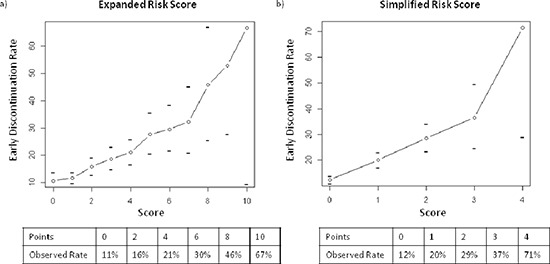
Relationship between model predicted score and observed early discontinuation rate, derivation set The line represents the total score [**(A)** expanded risk score, **(B)** simplified risk score] (x-axis) matched to the observed probability of early discontinuation (y-axis). Horizontal tick marks represents the 95% confidence interval around each estimate. The tables show the observed early discontinuation rate for selected scores.

**Figure 3:**
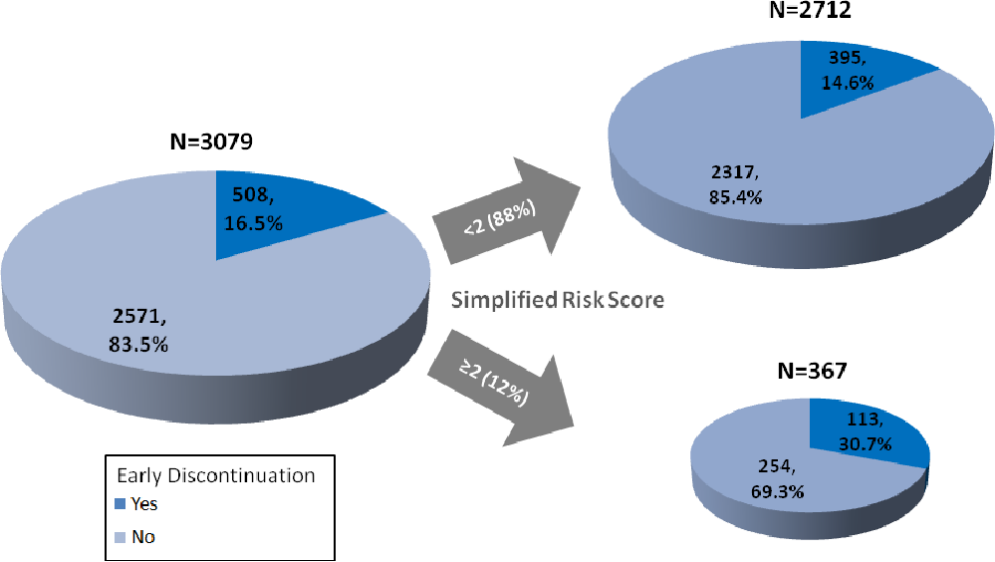
Impact of risk score on enrollment and early discontinuation This figure demonstrates the impact on the derivation set of excluding patients with a simplified risk score ≥ 2.

Figure 2 demonstrates the relationship of increasing points for the expanded and simplified risk scores to the observed early discontinuation rate in the derivation set. The performance of proposed cutoffs for both risk scores are presented in Table 3. In the derivation set, patients with ≥ 5 points on the expanded risk score or ≥ 2 points on the simplified risk score had approximately twice the observed rate of early discontinuation compared to patients with lower scores (31.6% vs 14.4% and 30.8% vs 14.6%, respectively, Figure 3). Patients with ≥ 3 points on the simplified risk score had a 40% likelihood of early discontinuation. Using these same point cutoffs (5 and 2 for the expanded and simplified risk scores, respectively), the overall correct classification rates (OCCRs) for both risk scores were approximately 80% in both the derivation and validation sets. The performance of the risk scores in the subset of patients who received molecular targeted agents only is shown in Supplemental Table 3A.

## DISCUSSION

Utilizing individual patient records from a very large, multi-institutional, contemporary cohort of North American patients enrolled to Phase I trials, we identified baseline clinical characteristics independently associated with the risk of early trial discontinuation. To our knowledge, this is the first such analysis performed to date. We identified several factors independently associated with early trial discontinuation including higher ECOG PS, alkaline phosphatase, AST, and platelets and decreasing albumin, hemoglobin, and creatinine clearance. These results offer important insights to physicians charged with selecting appropriate patients for Phase I trials.

Many of the risk factors identified here have previously been shown to be prognostic in the Phase I patient population [21]. Among patients who discontinue treatment early for reasons other than DLT, 61% did so for clinical disease progression or death and 23% due to adverse events (both disease and drug-related). These rates are very consistent with data reported by the European Drug Development Network [7] and suggest that our large multi-center derivation cohort accurately reflects the contemporary Phase I population. These data also suggest that the population of patients who discontinue treatment early tend to have a poor prognosis. The overlap of prognostic factors for 90-day survival and early discontinuation, however, is not complete. For example, although lymphopenia (ALC < 0.5 × 10^9^/L) is frequently cited as a prognostic factor, it did not predict for early discontinuation after adjusting for other covariates. Creatinine clearance, an independent predictor of early discontinuation, is not a well established prognostic factor. Moreover, the likelihood of early discontinuation due to progression or death was similar in our overall population and for patients with ≥ 2 points on our simplified risk score (see Supplemental Table 4A). These data suggest that patients who discontinue treatment early are not always those with the poorest prognosis and that excluding patients with poor prognosis does not eliminate early treatment discontinuation.

Using insight provided from the multivariate analysis, we created and externally validated two risk scores to identify patients at significantly increased risk for early discontinuation prior to enrollment. To arrive at this simplified risk score, we chose the risk factors with the greatest effect on early discontinuation (ECOG PS ≥ 2, albumin ≤ 3.5 mg/dL, alkaline phosphatase ≥ 2.5 x ULN, and hemoglobin ≤ 10 mg/dL). Patients with ≥ 2 of these risk factors prior to enrollment had an observed rate of early discontinuation of 31%, approximately twice the rate of the overall population (16.5%). By comparison, patients with none of these risk factors had only a 12% chance of discontinuing early, a relative risk reduction of 27% compared to the overall population. Both risk scores had similar performance in the derivation and validation sets, suggesting they are generalizable to new patient populations. Additionally, the risk scores performed similarly when assessed in the subset of patients who received molecular targeted agents only, indicating that these scores will continue to be relevant as drug development becomes more focused on these agents.

To illustrate how using the simplified risk score would impact patient selection and the composition of Phase I trials, Figure 3 shows the results of limiting accrual in the derivation cohort to patients with < 2 points on the simplified risk score. Enrollment of 11.9% (367/3079) patients would be curtailed by applying a cutoff of ≥ 2 points on the simplified risk score. The rate of early discontinuation in the remaining patients would be 14.6% (395/2712), compared to 16.5% (508/3079) in the original derivation set. In total, 113 fewer patients would discontinue early and 22.2% (113/508) of all early discontinuations would be avoided at the expense of curtailing enrollment by 367 patients. This cutoff would improperly exclude only 10% of those who do not discontinue early at the expense of failing to identify 78% of the patients who do discontinue early. This cutoff was chosen to minimize the impact on the overall pool of Phase I eligible patients while still providing a decrease in the number of patients who discontinue early. However, it is important to note that this cutoff would also improperly exclude 7 patients for every 3 patients accurately excluded. This “false positive” rate represents an important obstacle to the use of these scores in routine clinical practice. Even if these risk scores were implemented, early discontinuation rates would remain > 10%. It is therefore important that Phase I study sponsors account for this potentially unavoidable feature of the Phase I patient population when designing and conducting these studies. Study designs that minimize the need for delays in patient enrollment and dose escalation when one or more patients are inevaluable for toxicity due to early treatment discontinuation offer potentially significant advantages in the conduct of these studies [23].

Unfortunately, the number of patients seeking Phase I trials often exceeds study availability at high volume centers. As such, physicians often must select from multiple potentially eligible patients for a limited number of study spots. In doing so, physicians attempt to identify which patients are most likely to remain on study long enough to potentially benefit. Currently, physicians must rely solely on clinical experience to make these difficult judgments. In these circumstances, we believe that even highly expert Phase I investigators can benefit from the knowledge of how a limited number of objective patient characteristics may increase the risk of early treatment discontinuation.

## SUPPLEMENTARY TABLES


