# Supplementary material for: Predictors of early treatment discontinuation in patients enrolled on Phase I oncology trials
Source: Oncotarget. 2015 Feb 17;6(22):19316–27. doi: 10.18632/oncotarget.2909 (PMC4662493; doi:10.18632/oncotarget.2909)
Supplement: Supplementary file 1 [file oncotarget-06-19316-s001.pdf]

SUPPLEMENTARY TABLES

Supplementary Table S1A: Final multinomial multivariate model

Factor	OR (95% CI) – Early Discontinuation[1]	*P* value	OR (95% CI) – DLT[1]	*P* value
ECOG				
0	Ref		Ref	
1	1.30 (1.02–1.65)	0.0315	1.19 (0.98–1.45)	0.0866
≥ 2	2.22 (1.40–3.53)	0.0007	2.15 (1.42–3.25)	0.0003
Albumin (g/dL)				
< 3.5	1.59 (1.26–2.00)	< .0001	1.48 (1.20–1.82)	0.0002
≥ 3.5	Ref		Ref	
Alkaline Phosphatase (units/L)				
≤ 2.5xULN	Ref		Ref	
> 2.5xULN	1.62 (1.18–2.22)	0.0026	1.35 (1.00–1.81)	0.0484
AST (units/L)				
≤ULN	Ref		Ref	
>ULN	1.39 (1.09–1.77)	0.0076	1.26 (1.02–1.57)	0.0304
Creatinine clearance (ml/min)				
< 60	1.35 (1.01–1.81)	0.0455	1.33 (1.03–1.72)	0.0299
≥ 60	Ref		Ref	
Hemoglobin (g/dL)				
< 10	1.99 (1.46–2.71)	< .0001	0.81 (0.59–1.17)	0.2920
≥ 10	Ref		Ref	
Platelets (10^9/L)				
< 400	Ref		Ref	
≥ 400	1.30 (0.98–1.72)	0.0732	0.99 (0.75–1.30)	0.9336

1- Reference outcome: Continuation to Cycle 2.

Supplementary Table S2A: Comparison of risk factors in derivation and validation sets

Risk Factor	Derivation Set (*n* = 3079) N (%)	Validation Set (*n* = 232) N (%)
ECOG 1	2040 (66%)	158 (68%)
ECOG ≥ 2	154 (5%)	1 (0%)
Albumin (g/dL) < 3.5	802 (26%)	61 (26%)
Alkaline Phosphatase (units/L) ≥ 2.5xULN	355 (12%)	10 (4%)
AST (units/L) > ULN	788 (26%)	37 (16%)
Creatinine Clearance (ml/min) < 60	380 (12%)	36 (16%)
Hemoglobin (g/dL) < 10	273 (9%)	20 (9%)
Plates(10^9/L) ≥ 400	405 (13%)	21 (9%)
**Outcome**		
Early Discontinuation	508 (17%)	34 (15%)
Dose Limiting Toxicity	721 (23%)	31 (13%)
Cycle 2+	1850 (60%)	167 (72%)
**Type of experimental drug**		
MTA	1333 (43)	163 (70)
Cytotoxics	569 (18)	18 (8)
Cytotoxics and MTA	1177 (38)	51 (22)
**Number of Risk Factors**		
Number of Risk Factors[1] (median, range)	1, 0–7	1, 0–6
Number of Risk Factors[1]		
0	452 (15%)	41 (18%)
1	1139 (37%)	93 (40%)
2	774 (25%)	62 (27%)
3	451 (15%)	23 (10%)
4	175 (6%)	9 (4%)
5	73 (2%)	2 (1%)
6	13 (0%)	2 (1%)
7	2 (0%)	0 (0%)
**Full Multivariate Model**		
Model-predicted risk(median, range)	14.4% (10.0–56.9)	12.2% (10.0–50.5)
**Simplified Risk Score[2]**		
Number of Points		
0	1939 (63%)	162 (70%)
1	773 (25%)	50 (22%)
2	297 (10%)	18 (8%)
3	63 (2%)	2 (1%)
4	7 (0%)	0 (0%)

1- Counts ECOG 1 and ECOG ≥ 2 as a single risk factor.

2- Simplified Risk Score: ECOG 2, Alkaline Phosphatase ≥ 2.5xULN, Hemoglobin ≤ 10, and Albumin ≤ 3.5.

Supplementary Table S3A: Diagnostic accuracy of risk scores in the subset of patients receiving molecular agents only

Expanded Risk Score[1]

2 Points Each - ECOG 2, Alkaline Phosphatase ≥ 2.5xULN, Hemoglobin ≤ 10, and Albumin ≤ 3.5

1 Point Each - ECOG 1, Creatinine Clearance ≤ 60, AST ≥ ULN, Platelets ≥ 400

Simplified Risk Score[2] **2.5xULN , Hemoglobin ≤ 10, and Albumin ≤ 3.5**

		Derivation Set N = 1333	Validation Set N = 163
**Expanded Risk Score**: < 5 points vs ≥ 5 points	Sensitivity	59/256 = 23.1%	3/28 = 10.7%
	Specificity	991/1077 = 92.0%	124/135 = 91.9%
	OCCR	78.8%	77.9%
**Simplified Risk Score**: < 2 points vs ≥ 2 points	Sensitivity	56/256 = 21.9%	3/28 = 10.7%
	Specificity	996/1077 = 92.5%	125/135 = 92.3%
	OCCR	78.9%	78.5%

1- Maximum possible score: 11
2- Maximum possible score: 4
OCCR: Overall Correct Classification Rate.

Supplementary Table S4A: Reasons for early trial discontinuation, derivation set

	Total Early Discontinuations	Progression or Death N (%)	Toxicity (Non-Dose-Limiting) N (%)	Other N (%)
Overall Population	508	312 (61%)	115 (23%)	81 (16%)
Simplified Risk Score (< 2 points)	395	235 (59%)	93 (24%)	67 (17%)
Simplified Risk Score (≥ 2 points)	113	77 (68%)	22 (20%)	14 (12%)
